# WSV056 Inhibits Shrimp Nitric Oxide Synthase Activity by Downregulating *Litopenaeus vannamei* Sepiapterin Reductase to Promote White Spot Syndrome Virus Replication

**DOI:** 10.3389/fmicb.2021.796049

**Published:** 2021-12-23

**Authors:** Wei Wang, Qin Zheng, Chen Yu, Changkun Pan, Peng Luo, Jianming Chen

**Affiliations:** ^1^Institute of Oceanography, Minjiang University, Fuzhou, China; ^2^State Key Laboratory of Marine Environmental Science, College of Ocean and Earth Sciences, Xiamen University, Xiamen, China; ^3^State Key Laboratory of Biotherapy and Cancer Center, West China Hospital, Sichuan University, Chengdu, China; ^4^CAS Key Laboratory of Tropical Marine Bio-Resources and Ecology (LMB), Guangdong Provincial Key Laboratory of Applied Marine Biology, South China Sea Institute of Oceanology, Chinese Academy of Sciences, Guangzhou, China

**Keywords:** *Litopenaeus vannamei*, nitric oxide production, sepiapterin reductase, white spot syndrome virus, innate immunity, immediate-early protein, nitric oxide synthase, viral immune evasion

## Abstract

Sepiapterin reductase (Spr) plays an essential role in the biosynthesis of tetrahydrobiopterin (BH4), a key cofactor of multiple enzymes involved in various physiological and immune processes. Suppression of Spr could result in BH4 deficiency-caused diseases in human and murine models. However, information on the biological function of Spr in invertebrates is limited. In this study, two Sprs (CG12116 and Sptr) from *Drosophila melanogaster* were found to be downregulated in transgenic flies overexpressing white spot syndrome virus (WSSV) immediate-early protein WSV056. CG12116 and Sptr exerted an inhibitory effect on the replication of the *Drosophila* C virus. A *Litopenaeus vannamei* Spr (LvSpr) exhibiting similarity of 64.1–67.5% and 57.3–62.2% to that of invertebrate and vertebrate Sprs, respectively, were cloned. *L. vannamei* challenged with WSSV revealed a significant decrease in LvSpr transcription and Spr activity in hemocytes. In addition, the BH4 co-factored nitric oxide synthase (Nos) activity in shrimp hemocytes was reduced in WSSV-infected and LvSpr knockdown shrimp, suggesting WSSV probably inhibits the LvNos activity through LvSpr downregulation to limit the production of nitric oxide (NO). Knockdown of LvSpr and LvNos caused the reduction in NO level in hemocytes and the increase of viral copy numbers in WSSV-infected shrimp. Supplementation of NO donor DETA/NO or double gene knockdown of WSV056 + LvSpr and WSV056 + LvNos recovered the NO production, whereas the WSSV copy numbers were decreased. Altogether, the findings demonstrated that LvSpr and LvNos could potentially inhibit WSSV. In turn, the virus has evolved to attenuate NO production via LvSpr suppression by WSV056, allowing evasion of host antiviral response to ensure efficient replication.

## Introduction

*Litopenaeus vannamei* (white leg shrimp) is the most abundant shrimp species in the world. The worldwide production of *L. vannamei* from aquaculture reached 4.97 million tons in 2018, accounting for 52.9% of the global crustacean production from aquaculture (FAO, 2020). However, the increase in large-scale intensive aquaculture systems and severe pollution of the environment has led to outbreaks of diseases caused by various pathogens. White spot syndrome virus (WSSV) is considered the most destructive pathogen in shrimp, leading to huge economic losses to the shrimp farming industry (Dey et al., 2020).

White spot syndrome virus is enveloped, rod-shaped, and approximately 250 × 100 nm in size. The genome consists of 300 kb double-stranded DNA that contains approximately 185 open reading frames (ORFs). The virus could infect a wide range of hosts, and it was found to contaminate a large population of wild marine crustaceans (Xu et al., 2021), thus enabling the easy spread of WSSV to aquaculture species and new geographical locations. After infection, WSSV could cause rapid shrimp mortality up to 100% within 3–10 days. Thus far, no effective treatment is available to restrict the disease.

As an invertebrate, shrimp lack an adaptive immune system and rely on the innate immune reactions consisting of humoral immunity and cellular immunity to combat viral invasions. WSSV has evolved multiple strategies to counteract or exploit host immune responses for its benefit. For example, viral protein WSSV453 was reported to interact with proPPAE2 and interfere with its activation to active PPAE2 and lead to the inhibition of melanization in *L. vannamei* (Sutthangkul et al., 2017). Viral protein kinase 1 (PK1) suppresses apoferritin’s ability to incorporate iron to counteract the host cell’s iron-withholding defense mechanism (Lin et al., 2015). Moreover, WSSV could modulate the host’s immune-related pathways for its gene expression and genome replication. The viral protein WSSV449 showing homology to NF-κB pathway component Tube could activate the NF-κB pathway to enhance the transcription of viral genes wsv069, wsv303, and wsv371, with κB sites in their promoter regions (Wang et al., 2011). In addition, WSV181 could promote viral proliferation by inhibiting the JAK/STAT pathway (Wang et al., 2019).

White spot syndrome virus is a crustacean virus. Although the genome of the *L. vannamei* has been decoded, permanent cell lines and genetic manipulation tools are not available in crustacean species, which has hindered the studies on the control of shrimp viruses. As an arthropod insect, the model organism *Drosophila melanogaster* is a close relative to the crustacean and has been proven to be a useful tool for genetic analysis of host-pathogen interaction and antiviral innate immunity. With advantages of rapid generation time, inexpensive laboratory maintenance, availability of mutants, and RNA interference (RNAi) lines, *D. melanogaster* provides a powerful and efficient system for studying viral diseases. In addition, many innate immune responses against viruses in *Drosophila* are highly conserved in vertebrates, including JAK-STAT, IMD, and Toll pathways that trigger antimicrobial peptide (AMP) production, phagocytosis, and melanization to defend against viral infection.

In the previous study, transgenic fly lines, each expressing one WSSV protein, were constructed to characterize the function of WSSV proteins on the whole-organism level (Pan et al., 2017; Wang et al., 2019). Microarray data analysis in transgenic flies with or without WSV056 gene expression revealed sepiapterin reductase (Spr) gene CG12116 was most significantly inhibited by WSV056. Spr encodes the enzyme that catalyzes the final steps of tetrahydrobiopterin (BH4) biosynthesis. Thus far, two Spr genes have been identified from *Drosophila*. CG12116 and CG12117 (Sptr) are located in close vicinity to each other in the *Drosophila* genome. Homology modeling analysis suggested that the two genes have different substrate selectivity and different functions (Kim et al., 2015). BH4 is an essential coenzyme for nitric oxide synthase (Nos), along with three aromatic amino acid hydroxylases: phenylalanine-4-hydroxylase (Pah), tyrosine-3-hydroxylase (Th), and tryptophan-5-hydroxylase (Tph) (Yang et al., 2006). BH4 is pivotal for producing nitric oxide (NO) and monoamine neurotransmitters, including dopamine and serotonin from arginine, tyrosine, and tryptophan in animals (Vancassel et al., 2018). Loss of BH4 homeostasis could cause a range of problems related to the cardiovascular, nervous, and endocrine systems. For example, Spr inhibitors SPRi3 and N-acetylserotonin (NAS) could significantly reduce inflammatory pain. Another FDA-approved Spr-inhibiting drug, sulfasalazine, and polyamine inhibitor DFMO produces a synergistic antiproliferative effect on the tumorigenesis of neuroblastoma, an aggressive childhood malignancy (Yco et al., 2015). Spr gene-disrupted (*Spr^–/–^*) mice suffer from hypertension with fluctuation and bradycardia (Sumi-Ichinose et al., 2017). Spr has been a therapeutic target for various diseases. However, knowledge of the biological function of Spr has been focused on mammals, and the mechanism of Spr regulation is still to be elucidated.

The present study investigated the role of WSSV immediate-early protein WSV056 on regulating host immune response with the help of the *Drosophila* model system. We present evidence showing the overexpression of WSV056 facilitates the downregulation of two Sprs, including Sptr and CG12116, in transgenic flies. Spr deficiency in Sptr and CG12116 knockdown-S2 cells and fly lines compromised the host’s ability to resist *Drosophila* C virus (DCV) infection. Then we used *L. vannamei*, the native host of WSSV, to explore the underlying mechanism of the modulation of the host defense system by WSV056. Based on the reported investigation, *D. melanogaster* Spr genes (CG12116 and Sptr) and their *L. vannamei* homolog (LvSpr) play a role in the defense against viral infection of DCV and WSSV, respectively. Based on the investigation on the transcriptional regulation of LvSpr and the identification enzyme influenced by LvSpr with antiviral activity, WSSV may have the ability to inhibit Nos activity through transcriptional inhibition of LvSpr by WSV056. The results of this study provide a new perspective on the interaction between WSSV and host immune response.

## Materials and Methods

### Experimental Animals

All flies were maintained at 25 ± 0.5°C and 80 ± 5% humidity under 12 h of light/dark cycles on cornmeal-sucrose-yeast agar media. *w*^1118^ flies were used as wild-type controls. Fly lines were obtained from the Bloomington Drosophila Stock Center (BDSC) and the Vienna Drosophila Resource Center (VDRC). The following stocks were used:

*y^1^ w^67*c*23^* (BDSC 6599),

*da-Gal4* (BDSC 5460),

*w*^1118^ (BDSC 3605),

*UAS-wsv056*,

*tub-GAL80*^ts^**,

*CG12116 RNAi* (VDRC 6498), and

*CG12117 RNAi* (VDRC 17017).

Pacific white shrimp *L. vannamei* of approximately 4–5 g body weight were bought from a commercial farm (Zhanjiang, Guangdong Province, China). Before the experiment, shrimp were acclimated for 1 week in aerated artificial seawater with water salinity, with temperature maintained at 29–31 g/L and 24–26°C. Shrimp were fed with commercial feed three times a day at the rate of 5% body weight. PCR with WSSV-specific primers (Table 1) was performed to ensure that the shrimp were free of WSSV before experiments.

**TABLE 1:** Primers were used in this study.

Primers	Primer Sequences (5′–3′)
**cDNA clone**	
CG12116F	ATGGCAGCAAAGAGAATGGATT
CG12116R	GTCATAAAACGAAGGTGTCGTAATAGT
SptrF	ATGGACCTGAAACAGCGCAC
SptrR	GGAACTGCTCATCCCTGTAATCCA
LvSprF	GGCAAGCAAGACTGACAT
LvSprR	AACGGAACGAAGACCATC
LvSprGSP2	TGGCTCCAAGTGCGGCAATAGAGGTGATA
LvSprNGSP2	CTGTGTGCAGTGCAGCCCTTCAAGTCAT
LvSprGSP1	CTTGAAGGGCTGCACTGCACACAGAGAC
LvSprNGSP1	ATTGCCGCACTTGGAGCCAGTGATCGTA
**dsRNA template amplification**	
dsCG12116-F	GGATCCTAATACGACTCACTATAGGAATCGAAGCACTTGGAGCGA
dsCG12116-R	GGATCCTAATACGACTCACTATAGGGATCAGCTTGAGTGTGGCCT
dsSptr-F	GGATCCTAATACGACTCACTATAGGCGTGCAAACCTATTCGCTGG
dsSptr-R	GGATCCTAATACGACTCACTATAGGAAGTACATCTCACGGGCTGC
dsLvSpr-F	GGATCCTAATACGACTCACTATAGGCGAATGTGCCAAGTCCC
dsLvSpr-R	GGATCCTAATACGACTCACTATAGGTCAGCAGCCAAGACCTTAA
dsEGFP-F	GGATCCTAATACGACTCACTATAGGTCAGCGTGTCCGGCGAG
dsEGFP-R	GGATCCTAATACGACTCACTATAGG TCTTCTGCTTGTCGGCC
dsWSV056-F	GGATCCTAATACGACTCACTATAGGTTGGCGTATTTCTTCCC
dsWSV056-R	GGATCCTAATACGACTCACTATAGGCCACATTGGCATTCATTC
**Subcellular localization**	
pEGFP-CG12116-FF	GACTCAGATCTCGAGATGGCAGCAAAGAGAATGGATT
pEGFP-CG12116-R	CGACTGCAGAATTCGTCATAAAACGAAGGTGTCGTAATAGT
pEGFP-Sptr-F	GACTCAGATCTCGAGATGGACCTGAAACAGCGCAC
pEGFP-Sptr-R	CGACTGCAGAATTCGGAACTGCTCATCCCTGTAATCCA
**Dual-luciferase reporter assay**	
pCMV-HA-WSV056-F	CGGAATTCGGATGGCCTCAGTCTTTGAAGA
pCMV-HA-WSV056-R	GGGGTACCTTATTGTACCAAAAACTCAGAAATC
pGL3-CG12116P-F	ATAGGTACCGAGCTCCAAAAGGGGCTTGAAAAAATTAG
pGL3-CG12116P-R	CGGAATGCCAAGCTTTATTTTGATTTAAAGGATTTCGGGT
pGL3-SptrP-F	ATAGGTACCGAGCTCTTTCGCATCGAGTGAAGCTTAA
pGL3-SptrP-R	CGGAATGCCAAGCTTGATTGCGTGAAAAACACGGAC
**Real-time quantitative PCR**	
Rp49-qF	ATGACCATCCGCCCAGCATAC
Rp49-qR	ATGACCATCCGCCCAGCATAC
CG12116-qF	TTCAGTTCCCGAAATCCTCAA
CG12116-qR	CAGGGATTGACCCAAAGGATT
LvSpr-qF	TACGGTGTTGAACTATGCTCC
LvSpr-qR	AACAGATGTGCTACACGGAAG
Sptr-qF	ATCAAAGCCGAGGGTTCCAT
Sptr-qR	ATCAAAGCCGAGGGTTCCAT
WSV056-qF	GGCAAAGTTCCTACCCG
WSV056-qR	TGCTTCAAATGCTCCAC
WSSV-qF	AGTTGGCACCTTTGTGTGTGGTA qWSSV-R TTTCCACCGGCGGTAGCT
WSSV-qR	TTTCCACCGGCGGTAGCT
Lvβ-actin-qF	AGGCTAACCGCGAGAAGATGAC
Lvβ-actin-qR	GTAGCACAGTTTCTCCTTGATG

### Viral Infection

To determine the infectivity of the virus, DCV was titrated on S2 cell culture, and the Tissue Culture Infective Dose 50 (TCID_50_) was calculated using endpoint dilution by the method of Reed and Muench (1938). Adult flies of 3–5 days old were infected with DCV by intrathoracic injection as previously described (Wu et al., 2014). In brief, a thin needle dipped in DCV suspension (10^9^ TCID_50_ prepared in 10 mM Tris–HCl, pH 7.5) or control solution (10 mM Tris–HCl, pH 7.5) was inserted into the thorax of flies.

White spot syndrome virus challenge experiment was conducted on *L. vannamei* as described by Qiu et al. (2017). In brief, each shrimp in the experimental group was intramuscularly injected with approximately 1.0 × 10^6^ copies of WSSV in a 50 μL physiological saline solution in the third abdominal segment. *L. vannamei* treated with 50 μL saline were used as the negative control.

For survival assays, 20 flies or shrimp were used per treatment, and their survival was checked daily. Survival rates were analyzed through Kaplan–Meier curves by using GraphPad Prism. *P* < 0.05 was considered statistically significant.

The WSSV infectivity titer was calculated using the method of Reed and Muench (1938). Due to the lack of cell culture and permanent cell lines of shrimp, *in vivo* titration in *L. vannamei* was carried out to determine the shrimp infectious dose 50% endpoint (SID_50_/mL) as described by Corteel et al. (2012). In brief, 12 shrimp in each of the 3 replicates were intramuscularly injected with 50 μL of a 10-fold serial dilution of samples. At 48 hpi, the proportion of infected shrimp at each dilution was determined by qRT-PCR analysis.

### Plasmid Construction

For subcellular localization, the ORFs of CG12116 and Sptr were amplified from genomic DNA (gDNA) purified from *Drosophila* S2 cells as the template by using primer pairs CG12116F/R and SptrF/R. The PCR amplifications were inserted into the pEGFP-N1 vector (Clontech) using *Xhol* and *Eco*RI restriction sites to yield pEGFP-CG12116 and pEGFP-Sptr vectors, which could be used to express the GFP-tagged CG12116 and Sptr proteins, respectively.

Firefly luciferase reporter plasmids (pGL3-CG12116-P and pGL3-Sptr-P) were constructed by inserting the promoter region of CG12116 and Sptr PCR amplified from S2 cell gDNA into the pGL3-Basic luciferase reporter vector (Promega, Madison, WI, United States) at the *Sac*I and *Hin*dIII restriction sites to analyze the roles of WSV056 in the regulation of CG12116 and Sptr. The fragment containing the ORF of WSV056 was PCR amplified from a pET30a-WSV056 plasmid (Wang et al., 2020) and subcloned into a pCMV-HA vector (Clontech, CA, United States). All of the plasmids were confirmed by DNA sequencing. The primers used for plasmid construction are given in Table 1.

### Quantitative Real-Time PCR

Total RNA was extracted with TRIzol reagent following the manufacturer’s instructions (Invitrogen, United States). One microgram of total RNA was reverse transcribed to cDNA using PrimeScript RT Reagent Kit with gDNA Eraser (TaKaRa, China). Quantitative PCR was performed in triplicate by using SYBR Premix Ex Taq™ II (TaKaRa, China), along with gene-specific primers (Table 1) on Quantagene q225 (Kubo Technology, China). The expression levels of the genes of interest were normalized against the internal control gene ribosomal protein 49 (rp49) from *Drosophila* and the β-actin from white shrimp using the 2^–*CT*^ method.

For qRT-PCR analysis, at least three independent replicates of 10 flies or hemocytes from three shrimp each were used for RNA extraction. Every treatment was composed of three replicates. In all cases, three independent replicates were performed in parallel. At least two additional trials, each with three biologically independent replicates, were performed simultaneously, with similar results.

### Cells and Transfection

*Drosophila* S2 cells were cultured at 25°C in SF900-II SFM medium (Gibco, MA, United States) supplemented with 10% fetal bovine serum (FBS, Gibco, Grand Island, NY, United States) and penicillin/streptomycin (100 × mix, 10 mg/mL/10000 U, Invitrogen, MA, United States). 293T cells were cultivated in Dulbecco’s modified Eagle’s medium (DMEM) (Gibco, MA, United States) supplemented with 10% FBS at 37°C under humidified air containing 5% CO_2_.

Next, 293T cells were transiently transfected with the expression plasmids of interest using polyethylenimine (PEI, PolySciences Inc., Warrington, United States) as previously described (Gardner and Glaunsinger, 2018). In brief, cells growing at an exponential stage were seeded onto 6 cm dish cell plates the day before transfection. On the day of transfection, the cells were grown to 50–70% confluency. The medium was removed and replaced with a serum-free medium. DNA-PEI complexes were prepared in Eppendorf tubes containing 240 μL of DNA (32 μg/mL) and 180 μl of sterile PEI (250 μg/mL). After a brief vortex, the complexes were incubated at room temperature for 30 min and added dropwise to cells. After 6 h of incubation with the DNA–PEI complexes in the CO_2_ incubator (5% CO_2_), the medium was replaced by fresh FBS complemented with DMEM medium, and the incubation was continued for a total of 48 h.

### Subcellular Localization Analysis

For subcellular localization assay, 293T cells were seeded onto coverslips in cell culture dishes for 12 h. Then, they were transfected with pEGFP-CG12116, pEGFP-Sptr, or pEGFP-N1 (vector control). At 24 h post-transfection, the cells were incubated with Hoechst 33258 nucleic acid stain (Beyotime, China) for 30 min and washed with PBS. The fluorescent images were captured under a Leica TCS SP5 confocal laser scanning microscope (Heidelberg, Germany).

### Dual-Luciferase Reporter Assay

Firefly luciferase reporter plasmids (pGL3-CG12116P or pGL3-SptrP) were co-transfected with either a mixture of pRL-TK Renilla luciferase vector (control of transfection efficiency) and expression vector (pCMV-HA-WSV056) or a mixture of pRL-TK Renilla luciferase vector and the negative control vector (pCMV-HA) into 293T cells plated in 24 well plates to explore the regulatory effect of WSV056 on the promoter activity of CG12116 and Sptr genes. Cell lysates were harvested at 24 h post-transfection, and promoter activities were assessed using a Dual-luciferase Assay System (Promega, WI, United States) following the manufacturer’s instructions. Renilla luciferase activity was used to normalize the promoter activity between samples. All experiments were performed in three independent experiments, and each was analyzed in triplicate.

### Cloning of Full-Length cDNA of *Litopenaeus vannamei* Sepiapterin Reductase

The partial cDNA sequence of sepiapterin reductase was obtained from transcriptomic sequencing of *L. vannamei* by TBLASTN search of the NCBI database. The specific primer pair (LvSprF/R, Table 1) was designed to amplify the gene fragment from white shrimp hemocytes cDNA. In addition, 3’ and 5’ rapid amplification of cDNA ends (RACE) PCR was carried out using the SMARTer RACE kit (Clontech, Japan), following the user manual with the nested primers listed in Table 1. The PCR products were purified using a Gel Extraction Kit (Tiangen, China) and cloned into the pMD-18T vector (TaKaRa). The plasmids were transformed into competent *E. coli* DH5α cells, and positive colonies were screened by colony PCR and then sequenced. The full-length LvSpr cDNA was obtained by assembling the overlapping sequences.

### Sequence and Phylogenetic Analyses of *Litopenaeus vannamei* Sepiapterin Reductase

The full cDNA sequence of LvSpr was analyzed with EditSeq in DNAStar software (DNASTAR Inc., United States) to identify the ORF and translate it into amino acids. Conserved protein domains were predicted using the SMART program^1^. The molecular weight and theoretical isoelectric point were predicted using Compute pI/Mw tool^2^. The Spr protein sequences from various species were retrieved from the UniProt database^3^ by searching for protein sequences similar to LvSpr using the Blastp algorithm. Spr homologs were aligned by the Clustal Omega tool^4^. A phylogenetic tree was created with the deduced amino acid sequences of Spr via the neighbor-joining method implemented in MEGA6.0 software (Tamura et al., 2013).

### Tissue Distribution of *Litopenaeus vannamei* Sepiapterin Reductase

Ten healthy shrimp were randomly selected and dissected to investigate the tissue distributions of Spr in *L. vannamei*. The expression levels in nine tissue samples, including the eyestalk, gill, heart, hemocyte, hepatopancreas, intestine, muscle, nerve cord, and stomach, were analyzed using qRT-PCR assay primers listed in Table 1. The hemolymph was withdrawn from the ventral sinus by using a 1 mL syringe containing the same volume of precooled anticoagulant solution (400 mM NaCl, 0.1 M glucose, 30 mM trisodium citrate, 26 mM citric acid, and 20 mM EDTA; pH 4.6), followed by centrifugation at 800 × *g* for 10 min at 4°C. The hemocyte pellet and the other collected tissues were immediately frozen in liquid nitrogen and homogenized in three volumes of TRIzol with a tissue grinder for gene expression analysis.

### Assays of BH4 Production and Enzymatic Activity in *Litopenaeus vannamei* Hemocytes

The hemocyte samples were centrifuged and resuspended in precooled 0.86% physiological saline to prepare 10% (w:v) homogenates for enzyme activity measurement. Soluble proteins were collected by centrifugation at 18,000 × *g* for 15 min at 4°C. After the precipitates were discarded, the hemocyte lysate supernatant (HLS) was used for the spectro-photometrical measurement of BH4 production and enzymatic activities of Nos, Th, and Spr. According to the manufacturer’s instructions, the enzyme-linked immunosorbent assay (ELISA) assay was used to detect the BH4 level (Boyan Biotech, China). For the expression of enzyme activity, the tissue protein concentration in each sample was estimated using the method of Lowry et al. (1951), with BSA as the standard.

The Spr activity was determined by monitoring the decrease in the absorbance at 420 nm based on the NADPH-dependent reduction in sepiapterin (Tang et al., 2017). The reaction solution contained 350 μL of 0.2 mM potassium phosphate buffer (pH 6.4), 0.2 μM NADPH, 0.1 μM sepiapterin, and HLS. The reaction was initiated by the addition of HLS at 30°C.

The Th activity was assayed following Shiman et al. (1971) procedure by measuring the L-DOPA formed from L-tyrosine as substrate. The reaction mixture contained 350 μL of 1.4 mM L-tyrosine in PBS buffer (pH 6.2), 10 μL HLS, and 40 mM β-mercaptoethanol. Incubation was carried out at 30°C for 25 min, and the reaction was stopped by adding 0.5 mL of 0.5 N HCl. The reaction mixture was added with 500 μL of freshly prepared nitrite molybdate reagent (10 g of sodium nitrite and 10 g of sodium molybdate in 100 mL water). After incubation was performed for 5 min, 0.5 mL of 2 N NaOH was added, and the solution was quickly mixed. The absorption was immediately examined at 510 nm.

Nitric oxide synthase activity assay was performed to measure NO as an indicator of Nos activity. A commercial kit (Nanjing Jiancheng Bioengineering Institute, Nanjing, China) was used to monitor NO production at the absorbance of 530 nm.

### RNA Interference Experiment

dsRNAs targeting CG12116, Sptr, LvSpr, LvNos, WSV056, and EGFP were synthesized from T7-promoter-flanked PCR products by *in vitro* transcription using a T7 transcription kit (Promega). The list of primers used and their sequence is presented in Table 1. After purification, the dsRNAs were assessed by NanoDrop 2000 (Thermo Fisher Scientific, United States) and then stored at −80°C.

CG12116 and Sptr in the S2 cell line were knocked down as previously described (Zhang Q. et al., 2015). In brief, S2 cells were incubated with a serum-free medium containing 10 μg/mL dsRNA (dsCG12116, dsSptr, or dsEGFP). Six h later, the medium was removed and replaced with a complete medium. After 1 day of dsRNA treatment, the cells were infected with DCV (MOI 1). Samples were collected at different times for qRT-PCR analysis.

*In vivo* gene knockdown of *L. vannamei* was carried out as described (Tseng et al., 2019), with minor modification. Approximately 7 μg of dsRNA of LvSpr, LvNos, WSV056, or EGFP (negative control) suspended in 50 μL saline was separately injected into each shrimp intramuscularly. For double gene knockdown, the dsRNAs of two genes were co-injected. Shrimp injected with saline were set as blank control. At 48 h after injection, each shrimp in different groups was injected with 100 μL of saline containing 7 μg of the same dsRNA and 1 × 106 copies WSSV. For dietary supplementation experiments, the shrimp were fed with a commercial diet supplemented with BH4 and NO donor diethylenetriamine (DETA/NO) at doses of 20 and 0.4 mg/kg day before WSSV infection. Hemolymph was collected before injection as an initial control and at 48 h after the second injection. Hemocytes and HLS were prepared to measure relative gene expression and enzyme activity, respectively. The pleopods from each group were sampled at 48 h following WSSV infection, as Yang et al. (2020) described, to detect WSSV copy numbers.

## Results

### CG12116 and Sptr Were Downregulated in Transgenic Flies Expressing WSV056

**FIGURE 1:**
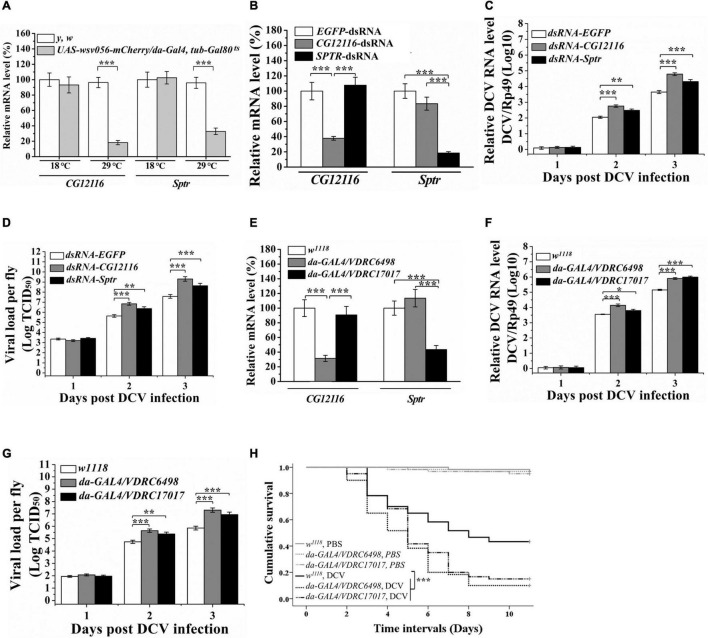
Knockdown of Sptr and CG12116 enhanced viral replication in DCV infected cultured cells and fly lines **(A)** RT-PCR analysis of the relative levels of *CG12116* and *Sptr* mRNA in *UAS-wsv056-mCherry/da-Gal4, tub-Gal80*^ts^** flies with WSV056-expression (raised at 18°C and temperature shifted to 29°C for 30 h before collection) and non-expression (raised at 18°C before collection). **(B)** The knockdown efficiency of CG12116 and Sptr genes in S2 cells treated with corresponding dsRNAs. The expression levels were normalized to the corresponding values expressed in the S2 cells treated with EGFP dsRNA. **(C,D)** DCV RNA accumulation **(C)** and viral titer **(D)** in S2 cells were determined by qRT-PCR analysis and endpoint dilution, respectively. **(E)** The transcriptional levels of CG12116 and Sptr in flies, respectively expressed CG12116-RNAi (VDRC 6498) or Sptr-RNAi (VDRC 17017). **(F,G)** DCV RNA accumulation **(F)** and viral titer **(G)** in CG12116 and Sptr knocked down fly lines and *w*^1118^ control fly line infected with DCV. **(H)** Kaplan-Meier curves show survival of CG12116 and Sptr knockdown flies and *w*^1118^ control flies following infection with DCV (black lines) or injection with PBS (gray lines). Differences in survival levels between the experimental and control groups were analyzed by Kaplan–Meier log-rank χ^2^ tests (*p < 0.05, **p < 0.01, and ***p < 0.001).

The Gal80*^ts^* TARGET system has been used for temporal regulation of WSV056 transgenic expression (Wang et al., 2020). The temperature-sensitive Gal80*^ts^* protein could bind to GAL4 at a permissive temperature of 18°C and antagonize Gal4 activity. At restrictive temperatures (25–29°C), the Gal80*^ts^*/Gal4 complex was dissociated to enable targeted expression of the UAS transgene under Gal4 control. Flies carrying transgenes of UAS-wsv056-mCherry/da-Gal4, tub-GAL80*^ts^* reared at 18°C and then shifted to 29°C for 30 h. Using mCherry as a reporter, the Gal4 transcriptional activity was determined to be turned on to allow targeted expression of the UAS transgene. In the second group, flies of the same line were raised at 18°C all the time to repress the expression of WSV056. Control (*y, w*) flies were subjected to the same temperature-shift program. Genetically identical flies with and without WSV056 expression were examined for the transcriptional levels of CG12116 and Sptr by qRT-PCR with primers displayed in Table 1. As shown in Figure 1A, CG12116 and Sptr were significantly downregulated in response to the overexpression of WSV056. No significant difference was observed in the gene expression levels in the *y, w* group and *UAS-wsv056-mCherry/da-Gal4, tub-Gal80*^ts^** group without a temperature-shift program.

### Knockdown of Sptr and CG12116 Enhanced *Drosophila* C Virus Replication in Cultured Cells

dsRNAs targeting Sptr, CG12116, or EGFP (non-targeting control) were generated to individually deplete them in *Drosophila* S2 cells via RNA interference (RNAi) to test the potential role of Sptr and CG12116 during viral infection. Effective knockdown of Sptr and CG12116 was confirmed by qRT-PCR analysis (Figure 1B). The RNAi-treated cells were subsequently challenged with *Drosophila* C virus, a natural pathogen of *Drosophila*. As shown in Figures 1C,D, the DCV titers and RNA levels in Sptr and CG12116-deficient cells increased compared with those in the control cells, suggesting viral RNA replication was promoted in Sptr- or CG12116-deficient cells.

### Knockdown of CG12116 or Sptr Increased Viral Replication and Mortality of *Drosophila* C Virus Infected Flies

The knockdown of *Drosophila* Sptr and CG12116 genes caused a high level of viral replication in S2 cells. Thus, whether knockdown of the two genes has the same effect on viral infection at the organismal level was further examined. Transgenic flies carrying the RNAi targeting Sptr (UAS-CG12117 RNAi, VDRC 17017) and CG12116 (UAS-CG12116 RNAi, VDRC 6498) were expressed under the ubiquitous driver da-Gal4. Efficiencies of these transgenes were validated by qRT-PCR. The results showed that endogenous Sptr and CG12116 in da-GAL4/VDRC 17017 and da-GAL4/VDRC 6498 flies significantly decreased compared with those in the control strain (Figure 1E), confirming the RNAi construct effectively knocked down Sptr and CG12116. The flies were then challenged with DCV, and the levels of DCV replication were monitored as a function of time post-infection. Figures 1F,G reveal a significant increase in viral production and infectivity in the Sptr and CG12116 knockdown flies. By contrast, the control and knockdown lines tolerated the control PBS injection to a similar degree (Figure 1H). In addition, the flies expressing decreased levels of Sptr and CG12116 were significantly more susceptible to DCV infection than the control flies (Figure 1H).

### CG12116 and Sptr Are Located in the Cytoplasm

**FIGURE 2:**
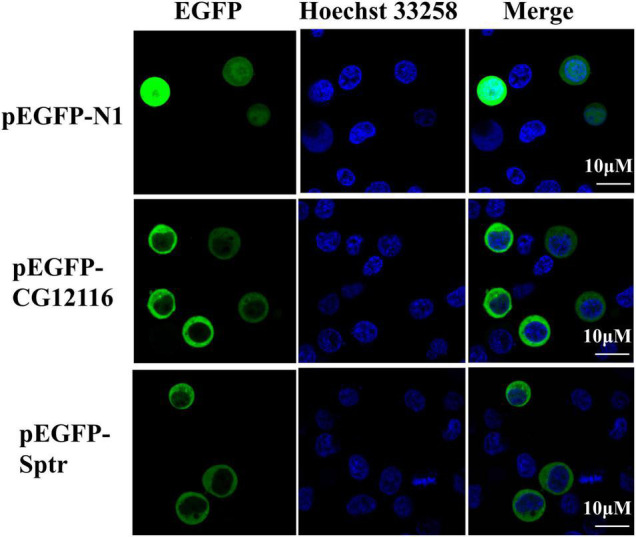
Subcellular localization of CG12116 and Sptr. The 293T cells were transfected with pEGFP-N1 (upper panels), pEGFP-CG12116 (middle panels), or pEGFP-Sptr (lower panels) expression plasmids. At 24 h post-transfection, the cells were stained with Hoechst 33258 and visualized with a confocal microscope. Green staining represents the signal of the EGFP-tagged proteins, and blue staining indicates the nucleus region.

Plasmids expressing EGFP fusion proteins with CG12116 and Sptr were constructed and transfected into 293T cells to identify the subcellular localization of CG12116 and Sptr. The confocal microscopy results (Figure 2) show that strong and homogeneous green fluorescence was detected in the cytoplasm in cells transferred with pEGFP-CG12116 and pEGFP-Sptr. In the control cells transfected with pEGFP-N1 empty vector, the fluorescent signal was observed in the nucleus and cytoplasm. The results suggested that CG12116 and Sptr were localized in the cytoplasm (Figure 2).

### WSV056 Protein Suppressed the Promoter Activity of CG12116 and Sptr

Dual-luciferase reporter assays were performed to assess the role of WSV056 on the transcriptional regulation of the promoter activity of CG12116 and Sptr. The assays revealed that the luciferase activities of pGL3-CG12116-P and pGL3-Sptr-P were significantly reduced by 36.3% (Supplementary Figure 1A) and 43.2% (Supplementary Figure 1B) in the 293T cells co-transfected with pCMV-HA-WSV056 compared with the cells co-transfected with pCMV-HA. These data suggested that WSV056 plays an essential role in suppressing the production of CG12116 and Sptr.

### Molecular Characterization of *Litopenaeus vannamei* Sepiapterin Reductase

A TBLASTN search of the NCBI database with *L. vannamei* as the selected species was carried out using the amino acid sequences of Sptr and CG12116 as the queries. No homology sequence to CG12116 was identified from the database of *L. vannamei* transcriptome and genome data. The partial cDNA sequence showing homology to Sptr was obtained from transcriptomic sequencing of *L. vannamei*. The Sptr ortholog gene was named as LvSpr. The complete LvSpr cDNA was cloned from the hemocytes of *L. vannamei* by PCR and RACE methods. The full-length sequence was 3,183 bp in length, and it contained a 75 bp 5’-untranslated region (UTR), a 2,307 bp 3’-UTR, and an 801 bp ORF that encoded a putative protein of 266 amino acid residues. The molecular mass was estimated to be 28.14 kDa, and the theoretical isoelectric point (pI) was 7.17. It does not contain a typical signal sequence or transmembrane domain. A SMART search revealed that the LvSpr protein contained an adh_short (short-chain dehydrogenase) domain between amino acids 11 and 219, indicating that LvSpr is a short-chain member dehydrogenases/reductases.

### Multiple Alignments and Phylogenetic Tree Analysis

The predicted amino acid sequences of Spr from *L. vannamei* were compared with the sequence of Spr from various species. Multiple sequence alignment showed that LvSpr displayed 64.1–67.5% and 57.3–62.2% similarity with Spr from invertebrate and vertebrate species, respectively.

The evolutionary relationship of the Spr sequences was analyzed with a phylogenetic tree constructed by the neighbor-joining method based on the deduced amino acid sequences. As shown in Supplementary Figure 2, LvSpr grouped with Spr from insect species to form an independent clade at the tree’s base, suggesting LvSpr has a closer evolutionary relationship with invertebrate Sprs than vertebrate Sprs. In vertebrate lineage, Spr proteins are further divided into four clades corresponding to bony fishes, amphibians, birds, and mammals, and they reflect the phylogeny of the chosen organisms. The presence of the two subgroups as TrSpr1/2 and DrSpra/b in the clade of bony fishes suggested that the duplication events occurred independently in the pufferfish and zebrafish lineages after they separated the common ancestor of fish species.

### Expression Levels of *Litopenaeus vannamei* Sepiapterin Reductase in Healthy and White Spot Syndrome Virus-Challenged Shrimp

Quantitative real-time PCR was employed to measure the relative expression levels of LvSpr mRNA in various tissues. As shown in Supplementary Figure 3, LvSpr was expressed in all the examined tissues. The highest transcript level was observed in hemocytes, moderate levels in the heart and hepatopancreas, and lower levels in the eyestalk gill, muscle, intestine, stomach, and nerve cord.

### Effect of White Spot Syndrome Virus Challenge on *Litopenaeus vannamei* Sepiapterin Reductase Expression Profile and Enzyme Activity in Hemocytes

**FIGURE 3:**
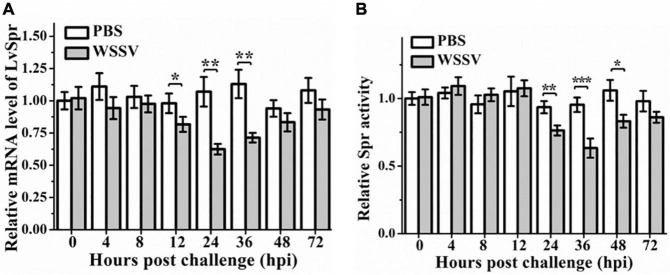
The expression profiles **(A)** and enzymatic activity **(B)** of LvSpr in hemocytes of *L. vannamei* post-challenge with WSSV. Hemocyte samples were collected at 0, 4, 8, 12, 24, 36, 48, and 72 h from shrimp challenged with 50 μL PBS containing 1.0 × 10^6^ copies of WSSV or 50 μL PBS as a control. Bars represented the mean ± SE (*n* = 3) from three experiments. The expression of LvSpr in hemocytes at 0 h of PBS challenged shrimp was set as 1.0. Asterisks indicate statistically significant differences of mRNA levels between the WSSV group and the corresponding PBS control group (*p < 0.05, **p < 0.01, ***p < 0.001).

*Litopenaeus vannamei* Spr showed the highest expression in hemocytes, one of the most important components of the shrimp immune system. Therefore, hemocyte was chosen to investigate the expression profiles of LvSpr after immune challenge with WSSV. As shown in Figure 3A, the transcriptional level of LvSpr was significantly downregulated from 12 to 36 hpi, and it reached the lowest level at 24 hpi and then recovered to the control level at 48 hpi. Meanwhile, no significant difference in the transcriptional levels of LvSpr was observed in the PBS-injected group. The effect of the WSSV challenge on LvSpr activity in *L. vannamei* hemocytes is shown in Figure 3B. Compared with the PBS-injected group, a significant reduction in LvSpr activity was observed from 24 to 48 h after stimulation with WSSV.

### BH4 Production and Nitric Oxide Synthase Activity Were Inhibited in White Spot Syndrome Virus-Infected and *Litopenaeus vannamei* Sepiapterin Reductase Knockdown Shrimp

**FIGURE 4:**
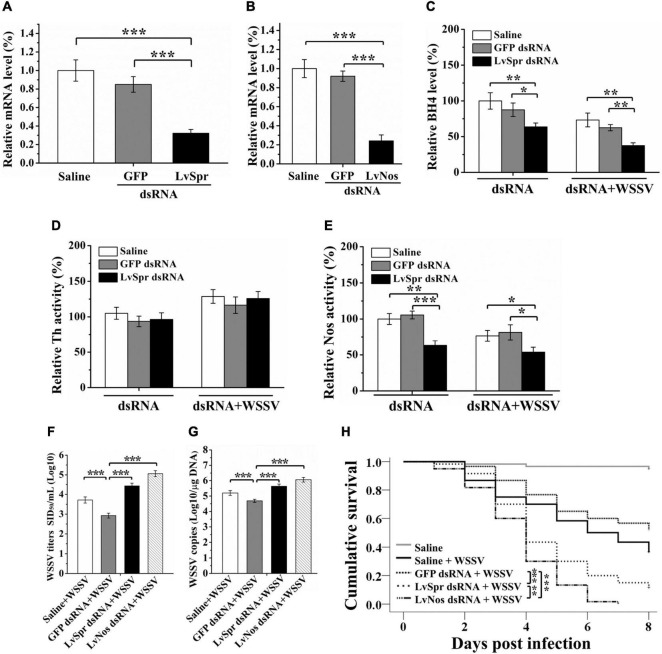
Regulation of BH4 production and BH4 dependent enzyme by LvSpr during WSSV infection. **(A,B)** qRT-PCR analysis of the RNA interference efficiency of LvSpr **(A)** and LvNos **(B)**. Shrimp were injected with LvSpr dsRNA, LvNos dsRNA, GFP dsRNA (negative control), and saline (blank control) twice in a 48 h interval. Hemolymph was collected at 48 h after the second dsRNA injection. **(C–E)** BH4 **(C)** level and the enzymatic activity of Th **(D)** and Nos **(E)** in the HLS of LvSpr knockdown shrimp. The dsRNAs were injected into shrimps twice in a 48 h interval. HLS was prepared to measure the BH4 level and enzymatic activity 48 h after the second injection of dsRNA (left panel) or 48 h after the second injection of dsRNA and WSSV virions (right panel). **(F,G)** WSSV load **(F)** and infection titer **(G)** in the LvSpr, LvNos, and GFP silenced shrimp as well assaline-injected shrimp infected with WSSV. **(H)** Kaplan-Meier curves showing survival of LvSpr, LvNos, and GFP-silenced and saline-injected shrimp following infection with WSSV. Differences in survival levels between the experimental and control groups were analyzed by Kaplan–Meier log-rank χ^2^ tests (asterisks indicate significant differences *p < 0.05, **P < 0.01, and ***P < 0.001).

Sepiapterin reductase plays a key role in the biosynthesis of BH4, the essential cofactor for multiple enzymes, including Pah, Th, Tph, and Nos. Among these enzymes, Th and Nos have been identified in *L. vannamei* (Yao et al., 2010; Mapanao and Cheng, 2016). *In vivo* knockdown of *LvSpr* in *L. vannamei* was performed via injection of dsRNA, followed by WSSV challenge, to investigate the potential functional role of LvSpr in the regulation BH4-dependent enzymes. Shrimp injected with GFP dsRNA or saline were set as negative control and blank control, respectively. Silencing efficiency was detected by qRT-PCR at 48 h after dsRNA treatment. As shown in Figure 4A, the LvSpr expression in shrimp hemocytes was significantly decreased. Meanwhile, no suppressive effect on the LvSpr mRNA level was observed in the GFP-dsRNA injected group.

Hemolymph BH4 level and the enzymatic activity of Th and Nos were measured in shrimp 48 h after dsRNA treatment. As shown in Figures 4C,E, the BH4 level and Nos activity were significantly decreased at 48 h after treatment of LvSpr dsRNA, WSSV, and LvSpr dsRNA plus WSSV, compared with the saline and GFP dsRNA injected control groups. In contrast, the Th activity level in the LvSpr knockdown group was not significantly different from that of the control groups injected with GFP dsRNA and saline (Figure 4D). The results suggested that LvSpr expression and WSSV infection have an inhibitory effect on BH4 production and NOS activity in shrimps hemocytes.

### Roles of *Litopenaeus vannamei* Sepiapterin Reductase and LvNos During White Spot Syndrome Virus Infection

dsRNA inference experiments were performed to investigate the potential influence of LvSpr and LvNos during WSSV infection. As shown in Figures 4A,B, the transcriptional level of LvSpr and LvNos in shrimp hemocytes from the specific dsRNA-injected groups decreased significantly compared with that from the saline- and GFP dsRNA-injected groups.

Having confirmed the gene knockdown efficiency of LvSpr and LvNos by their respective dsRNAs, an RNAi experiment was performed, followed by WSSV infection. Then, the WSSV replication and survival rate of shrimp were analyzed. At 48 h post-WSSV infection, the virus titers and virus loads were detected by intramuscular injection of shrimp and qRT-PCR (Figures 4F,G). Compared with the control groups injected with saline and GFP dsRNA, the titers and genome copies of WSSV were significantly increased in shrimp injected with LvSpr and LvNos dsRNA, suggesting that the RNAi of LvSpr and LvNos led to a significant promotion of WSSV replication. Consistent with the virus load results, the deficiency of LvSpr or LvNos resulted in a notably faster increase of cumulative mortality following WSSV infection (Figure 4H). The WSSV-infected shrimp pretreated with LvNos dsRNA succumbed to death after 7 days post-infection. The group pretreated with LvSpr dsRNA showed a marked increase in survival, with 11.7% shrimp remaining after 8 days post-infection. By contrast, the shrimp in the control groups challenged with WSSV alone or GFP dsRNA + WSSV exhibited significantly increased survival rates of 36.7 and 51.7%, respectively. The above findings indicated that LvSpr and LvNos could play positive roles in the host’s defense against WSSV infection.

### Functional Analysis of WSV056, *Litopenaeus vannamei* Sepiapterin Reductase, and LvNos in the Regulation of Host Innate Immunity by White Spot Syndrome Virus

**FIGURE 5:**
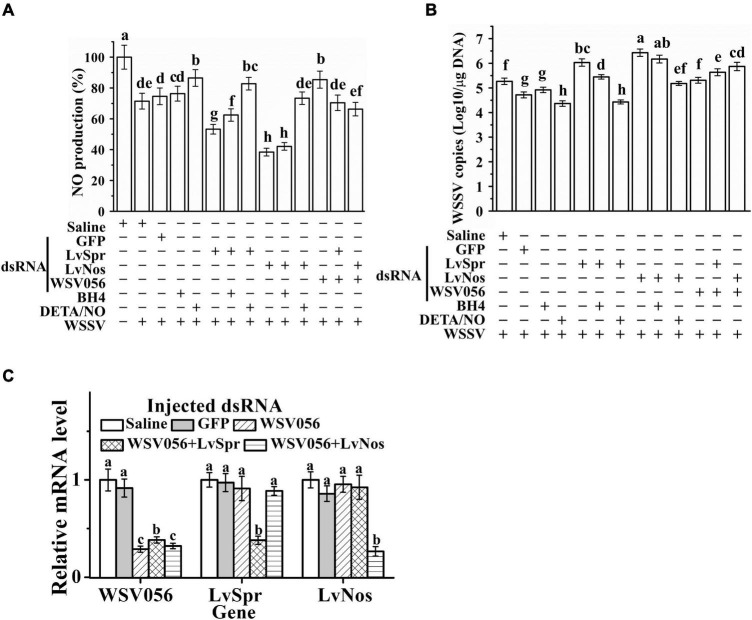
Roles of WSV056, LvSpr, and LvNos to regulating host innate immunity by WSSV. **(A,B)** NO production **(A)** and WSSV load **(B)** of single gene knockdown of LvSpr, LvNos, and WSV056 and double gene knockdown of WSV056 + LvSpr and WSV056 + LvNos. The dsRNAs were injected into shrimp twice in a 48 h interval. From 24 h after the first injection, shrimp started to be fed with BH4 or DETA/NO supplemented diet. Shrimp hemocytes were sampled at 48 h after the second injection of dsRNA and WSSV virions. Different letters (above each bar) indicate a significant difference (*P* < 0.05) in the mean NO production **(A)** or WSSV load **(B)** among the samples from different groups. **(C)** qRT-PCR analysis of the RNA interference efficiency of WSV056 in the group with single gene knockdown of WSV056 and double gene knockdown groups of WSV056 + LvSpr and WSV056 + LvNos infected with WSSV. Expression values were normalized to those of β-actin.

Sepiapterin reductase catalyzes the biosynthesis of BH4, which is the cofactor of Nos responsible for the production of NO. The effect of BH4 and NO donor DETA/NO on LvSpr and LvNos interfered shrimp was investigated to determine whether the enhanced WSSV replication in LvSpr and LvNos knockdown shrimp is related to the depletion of preexisting BH4 and NO. LvSpr or LvNos was knocked down by RNAi, and the NO production (as an indication of Nos activity) and WSSV titers were determined 48 h after WSSV infection. As shown in Figure 5A, a significant reduction in NO production was found in the shrimp interfered with LvSpr or LvNos. The reduction corresponded to the decreased Nos activity in WSSV-infected shrimp, suggesting that WSSV may suppress the NO production of the host. In addition, LvSpr and LvNos are likely to be the target enzymes suppressed by the virus.

Dietary supplementation of DETA/NO significantly reduced WSSV replication in the infected shrimp (Figure 5B). DETA/NO could also rescue NO production and WSSV inhibition in LvSpr and LvNos knockdown shrimp. By contrast, BH4 could only partially rescue NO production and WSSV inhibition in LvSpr knockdown shrimp. When the shrimp were treated with LvNos dsRNA, adding BH4 had no significant effect on NO production and viral titer. These results indicated that the inhibitory effect of DETA/NO on WSSV replication was due to the restoration of NO production inhibited by WSSV through the suppression of LvSpr and LvNos activities.

Single gene knockdown of WSV056 and double gene knockdown of LvSpr + WSV 056 and LvNos + WSV056 were performed to investigate the potential function of WSV056 in the suppression of shrimp NO activity (Figures 5A,B). The knockdown efficiency was checked by qRT-PCR at 48 h after WSSV infection (Figure 5C). The transcription level of WSV056 in the single gene knockdown group was significantly reduced compared with that in the saline- and GFP dsRNA-injected groups. Double gene knockdown was conducted by injecting the mixed dsRNA of two genes. In shrimp injected with a combination of LvSpr + WSV 056 or LvNos + WSV056 dsRNAs, a clear double knockdown of LvSpr/WSV056 and LvNos/WSV056 was found in both groups. Meanwhile, no significant difference in the transcription level of tested genes was observed in the GFP knockdown shrimps (Figure 5C).

As shown in Figure 5A, a comparison between the saline- and GFP dsRNA-injected control groups showed that the NO production was significantly increased in the WSV056 single gene knockdown group. However, double gene knockdown of LvSpr + WSV 056 or LvNos + WSV056 did not increase the NO production during WSSV infection, suggesting that the ability of WSV056 to inhibit NO production was related to the activity of LvSpr and LvNos. Interestingly, the LvSpr + WSV056 or LvNos + WSV056 double gene knockdown groups showed significantly higher NO production than the LvSpr or LvNos single gene knockdown group, consistent with the decrease in virus titers. The results suggested that WSV056 was involved in the suppression of NO production during WSSV infection. LvSpr and LvNos mediated the suppression that contributed to WSSV replication.

## Discussion

Upon infection, WSSV genes classified as immediate-early (IE), early, or late genes are expressed in a temporally ordered fashion. IE genes are the first viral genes expressed dependent solely upon the host cellular environment. IE genes usually encode regulatory proteins playing essential roles in regulating viral early and late gene expression. In addition, IE proteins play important roles in manipulating the host cellular environment and limiting host antiviral mechanisms (Lu et al., 2011). For example, the Epstein-Barr virus immediate-early protein BZLF1 inhibited the IFN-γ induction of MHC surface expression and STAT1 tyrosine phosphorylation (Morrison et al., 2001). In herpes simplex virus type 2, IE proteins US1 and ICP27 downmodulated IFN-β production by antagonizing the association of IRF3 with the IFN-β promoter and interfering with IRF3 phosphorylation, respectively (Zhang M. et al., 2015; Guan et al., 2019).

Among the 21 IE genes identified from WSSV, IE1 (WSV069) is the best-understood one. Studies on IE1 mainly focused on its ability to hijack the shrimp transcriptional factors to enhance viral infection. *L. vannamei* regulatory factors, including Stat (Yao et al., 2016), Relish and Dorsal (Qiu et al., 2014), KLF (Huang et al., 2014), Yin Yang 1 (Huang et al., 2017), c-Fos and c-Jun (Li et al., 2015), and SIRT1 (Kao et al., 2020), could significantly activate IE1 promoter. In addition, the TATA box-binding protein of black tiger shrimp *Penaeus monodon* (Liu et al., 2011) could interact with IE1 to enhance viral gene expression and help in virus replication. Moreover, IE1 and its paralog IE protein WSV056 could bind to shrimp retinoblastoma protein (Rb) to stimulate the G1/S transition of cells and enhance viral replication (Ran et al., 2013). WSV056 is highly homologous to IE1 in amino acid sequence. However, knowledge of the role of WSV056 in WSSV infection is comparatively limited.

In the previous study, a transgenic *Drosophila* model overexpression of WSV056 was established. *D.* melanogaster overexpressing WSV056 became resistant to DCV infection, evidenced in reduced mortality and viral loads, compared to the wild-type control. Microarray data analysis identified three serine proteases (SPs) significantly upregulated in the WSV056-expressing group. Functional analysis of known SPs in *L. vannamei* suggested that shrimp SP LvPPAE2 induced by WSV056 contribute to restricting viral replication by promoting antimicrobial peptides expression and hemolymph phenoloxidase activity. In addition, a range of immune-related genes was downregulated in WSV056-expressing flies. The Spr gene CG12116 was the most significantly inhibited by WSV056 (Wang et al., 2020). Consistent with the previous result, the present study showed that the transcription levels of CG12116 and its paralog Sptr were downregulated in response to ectopic WSV056 expression (Figure 1A). In addition, dual-luciferase reporter assay revealed that the two *Drosophila* Sprs’ promoter activity was inhibited by WSV056 (Supplementary Figure 1).

Furthermore, Sptr and CG12116 were found to play an antiviral role against DCV infection in S2 cells and flies. Knockdown of CG12116 and Sptr in S2 cells led to increased replication in DCV (Figure 1C). When transgenic flies carrying the RNAi targeting Sptr or CG12116 were infected with DCV, higher viral production and mortalities were observed (Figures 1F,H). These results suggested that WSV056 mediated promoter inhibition of CG12116 and Sptr, promoting DCV replication.

Sepiapterin reductase is essential for the biosynthesis of BH4, a key cofactor for a set of enzymes associated with various biological processes. Human Spr has been related to diseases, including brain dysfunction, chronic pain, cardiovascular disease, and cancer (reviewed by Wu et al., 2020). The function of Spr related to defense against viral infection is largely unknown. In invertebrates, Sprs has been identified with enzyme activity characterized from *D. melanogaster*, housefly *Musca domestica*, and silkworm *Bombyx mori* (Iino et al., 1996; Kim et al., 2015; Tang et al., 2017). Comparative transcriptome analysis of *L. vannamei* revealed that the Spr transcription level was significantly higher in shrimp with high feed efficiency (Dai et al., 2017). However, the biological function of these invertebrate Sprs remained largely unclear.

One Spr gene showing homology to Sptr from *L. vannamei* was cloned and characterized in the study to identify the potential function and regulation of Spr from the native host of WSSV. As Sprs detected from mammalian species, LvSpr was constitutively distributed in multiple tissues. The maximal level of LvSpr was observed in hemocytes (Supplementary Figure 3). The expression and activity levels of LvSpr decreased with the WSSV challenge (Figures 3A,B). Due to the essential role of Spr in BH4 synthesis, LvSpr is likely to regulate BH4-dependent enzymes to modulate shrimp antiviral response. Based on transcriptome and genomic data searching, two out of the four BH4 co-factored enzymes were found in *L. vannamei*. Th and Nos are closely associated with invertebrate innate immunity. The genes encoding *L. vannamei* Th and Nos have been cloned (Yao et al., 2010; Mapanao and Cheng, 2016). In the present study, the two enzymes’ activity was analyzed in shrimp treated with LvSpr dsRNA or LvSpr dsRNA + WSSV infection to investigate if these two genes were involved in Spr-mediated immune response. The result revealed that WSSV infection and LvSpr inhibition could reduce Nos activity, whereas the Th activity was unaffected by LvSpr (Figures 4D,E). Thus, Spr could be involved in WSSV-induced immune response through the regulation of enzymatic activity of LvNos.

Nitric oxide synthase is a critical enzyme that catalyzes the synthesis of NO, a molecule that functions to provide effective protection against bacterial and parasite infections. NO plays a role in decreasing parasite loads in Eastern oyster *Crassostrea virginica* at early time points after infection (Villamil et al., 2007). The transcriptional levels of Nos from Kuruma shrimp *Marsupenaeus japonicus* and mud crab *Scylla paramamosain* were significantly enhanced after challenge with pathogenic vibrios (Inada et al., 2010; Li et al., 2012). In *L. vannamei*, NO production induced by *V. harveyi* challenge functions as a bactericidal molecule for *Vibrio* clearance (Chen et al., 2015). Administration of the anti-Nos serum to *L. vannamei* leads to reduced clearance of *Aeromonas hydrophila* in the hemolymph (Rodríguez-Ramos et al., 2016), suggesting that Nos is required for the immune defense against Gram-negative bacteria. The role of NO or Nos in invertebrate viral infections has not been well defined yet. In the hemocytes of Chinese shrimp *Fenneropenaeus chinensis* and *M. japonicus*, Nos activity increased during the first 12 and 48 h post-WSSV challenge before a sharp decrease afterward. Mortality experiment showed *F. chinensis*, with Nos activity kept at a high level for a shorter time after WSSV infection, succumbed to death much faster than *M. japonicus* (Jiang et al., 2006), suggesting the anti-WSSV function of shrimp Nos. Similarly, the present study found that LvNos activity decreased at 48 h post-WSSV challenge (Figure 4E), and knockdown of LvNos enhanced viral load and mortality in *L. vannamei* infected with WSSV (Figures 4F,H). These results further verified the positive function of LvNos in the defense against WSSV infection in *L. vannamei*.

This study found that WSV056 could inhibit the transcription of *Drosophila* Sprs, including Sptr and CG12116 (Figure 1A). In *L. vannamei*, reduced Nos activity was found in WSSV-infected and LvSpr knockdown shrimp (Figure 4E). These results led to the hypothesis that the suppression of Spr transcription by WSV056 may somehow interfere with Nos activity and antiviral defense in *L. vannamei*. The reduced Nos activity in WSSV infected shrimp was partially rescued by WSV056 interference. However, the double knockdown of WSV056 and LvSpr failed to recover NOS activity (Figure 5). The results supported that WSSV056 impaired Nos activation via LvSpr.

Whether dietary supplementation of BH4 and NO donor DETA/NO could restore compromised anti-WSSV defense in LvSpr and LvNos knockdown shrimp was tested to identify the effect of LvSpr and LvNos in immune defense against WSSV. BH4 and DETA/NO showed therapeutic potential in the safe and efficient treatment of various diseases in animal models and human patients. The final step of BH4 biosynthesis is catalysis by Spr. Exogenous BH4 supplementation could rescue the cardiac and mitochondrial defects of Spr^–/–^ mouse and decrease diurnal tyrosine variations in patients with phenylketonuria caused by Pah deficiency (Kim et al., 2019; van Wegberg et al., 2021). DETA/NO is a long half-life diazeniumdiolate-class NO donor. NOS generated NO in the reaction requires a cofactor of BH4. Administration of DETA/NO could effectively reverse cerebral vasospasm resulting from NO depletion in dogs and enhance the host defense in mice subjected to hyperoxia accompanied by a decrease in the NO airway (Wolf et al., 1998; Gore et al., 2020). Previous studies indicated that 20 mg/kg/day BH4 and 0.4 mg/kg/day DETA/NO have a half-life of approximately 6 and 20 h, respectively (Keefer et al., 1996; Kör et al., 2017). The daily dose of BH4 was divided into three doses to the shrimp diet to prevent level fluctuations throughout the day. The lower NO production and WSSV inhibitory activity in shrimp treated with LvSpr and LvNos dsRNA could be restored by treatment of DETA/NO, thus suggesting that the inhibition of LvSpr and LvNos leads to the suppression of NO production and antiviral defense. BH4 supplementation could rescue LvSpr deficiency caused by abnormality but failed to rescue the effect caused by LvNos knockdown, indicating that the reduced level of NO production in LvNos knockdown shrimp is not associated with BH4 deficiency. Moreover, the results revealed the therapeutic potential of NO donors in the prevention and treatment of WSSV infection.

In summary, this study demonstrated the importance of BH4 synthesis and NO production in the defense against WSSV infection. By downregulating the transcription of LvSpr, WSV056 inhibited BH4 synthesis and resulted in the suppression of host NO production and an increase in WSSV copy number in WSSV-infected shrimp. These findings provide novel insights into the regulatory role of WSSV056 in attenuating host antiviral immune response, which leads to the promotion of viral replication in WSSV-infected shrimp. The research further enriched knowledge on invertebrate antiviral innate immunity and immune evasion mechanism devised by the virus.

## Data Availability Statement

The datasets presented in this study can be found in online repositories. The names of the repository/repositories and accession number(s) can be found below: https://www.ncbi.nlm.nih.gov/genbank/, OK490362.

## Ethics Statement

The animal study was reviewed and approved by the Institutional Animal Care and Use Committee of Minjiang University.

## Author Contributions

JC provided the experimental ideas and design of the study. QZ, CY, and PL carried out the experiments. WW and CP analyzed the data. WW and PL wrote the manuscript. All authors contributed to the article and approved the submitted version.

## Conflict of Interest

The authors declare that the research was conducted in the absence of any commercial or financial relationships that could be construed as a potential conflict of interest.

## Publisher’s Note

All claims expressed in this article are solely those of the authors and do not necessarily represent those of their affiliated organizations, or those of the publisher, the editors and the reviewers. Any product that may be evaluated in this article, or claim that may be made by its manufacturer, is not guaranteed or endorsed by the publisher.

## Footnotes

1
http://smart.embl-heidelberg.de/

2
https://www.expasy.org/resources/compute-pi-mw

3
http://www.uniprot.org/

4
http://www.ebi.ac.uk/Tools/msa/clustalo/
