# Supplementary material for: WSV056 Inhibits Shrimp Nitric Oxide Synthase Activity by Downregulating Litopenaeus vannamei Sepiapterin Reductase to Promote White Spot Syndrome Virus Replication
Source: Front Microbiol. 2021 Dec 23;12:796049. doi: 10.3389/fmicb.2021.796049 (PMC8733705; doi:10.3389/fmicb.2021.796049)
Supplement: Supplementary file 1 [file Data_Sheet_1.docx]

Fig S1Suppression of CG12116 and Sptr promoters by overexpression of WSV056. 293T cells were seeded in 24-well plates for 24 h and co-transfected with reporter plasmids pGL3-CG12116-P (a) or pGL3-SPTR-P (b), protein expression vectors pCMV-HA-WSV056 or pCMV-HA (empty vector as a control), and pRL-TK Renilla luciferase vector (as an internal control). The luciferase assays were performed 24 h after transfection. The data were expressed as mean ± standard deviation (n = 3), and the experiments were repeated three times with similar results. Asterisks indicate significant differences from control (****P*< 0.001).


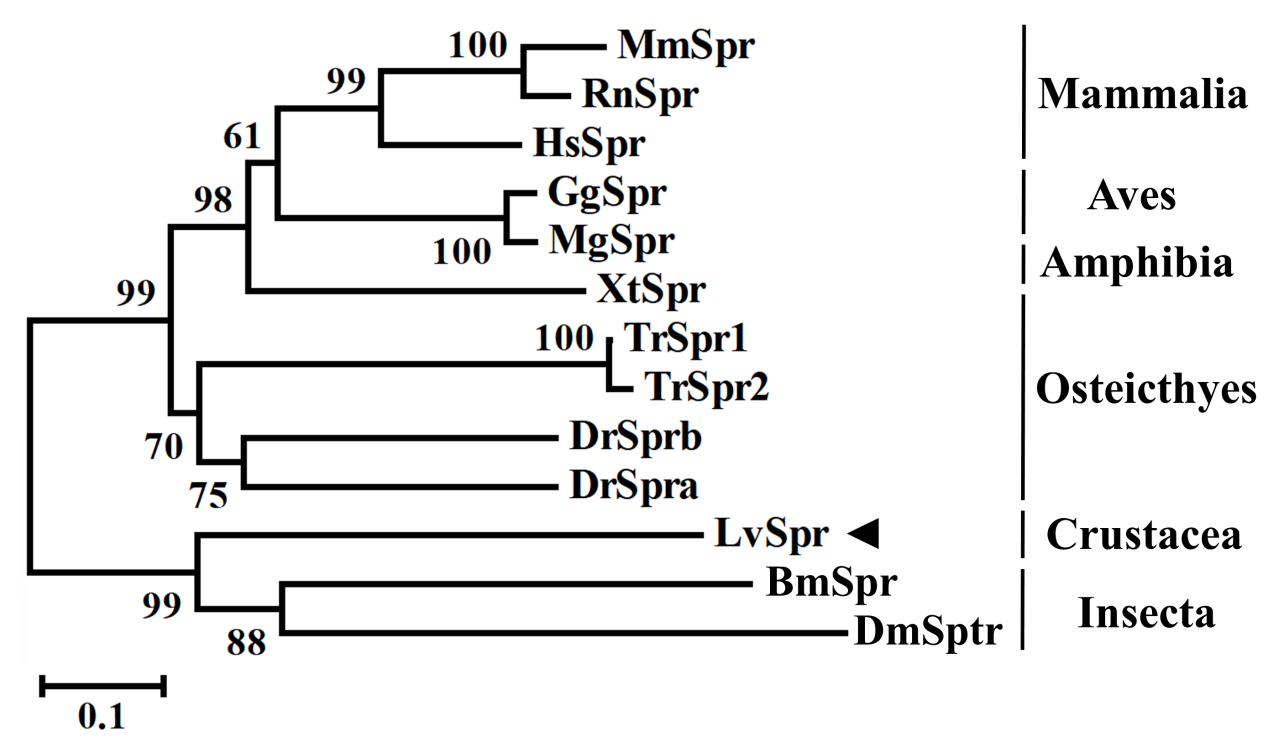


Fig. S2. Phylogenetic analysis of Spr proteins. The tree was deduced by neighbor-joining analysis based on the alignment of the amino acid sequences of Spr proteins by using MEGA4. The genes used are listed as follows, HsSpr, *Homo sapiens* Spr (Accession No. P35270); GgSpr, *Gallus gallus* Spr (Accession No. E1C4L3); MgSpr, *Meleagrisgallopavo* Spr (Accession No. G1NKT4); XtSpr, *Xenopustropicalis* Spr (Accession No. B0BML7); TrSpr1,*Takifugurubripes* Spr1 (Accession No. H2US29); TrSpr2, *Takifugurubripes* Spr2 (Accession No. O42496); DrSpra, *Daniorerio*Spra (Accession No. F1QWX4); DrSprb, *Daniorerio*Sprb (Accession No. B3DJY2); BmSpr, *Bombyxmori*Spr (Accession No. C0STP5); DmSptr, *Drosophila melanogaster* Sptr (Accession No. O76752). Numbers at the nodes indicated the levels of bootstrap support based on data for 1000 replicates. Only values greater than 50% were shown. LvSpr was marked with solid triangle. The Bar (0.1) shows the genetic distance.


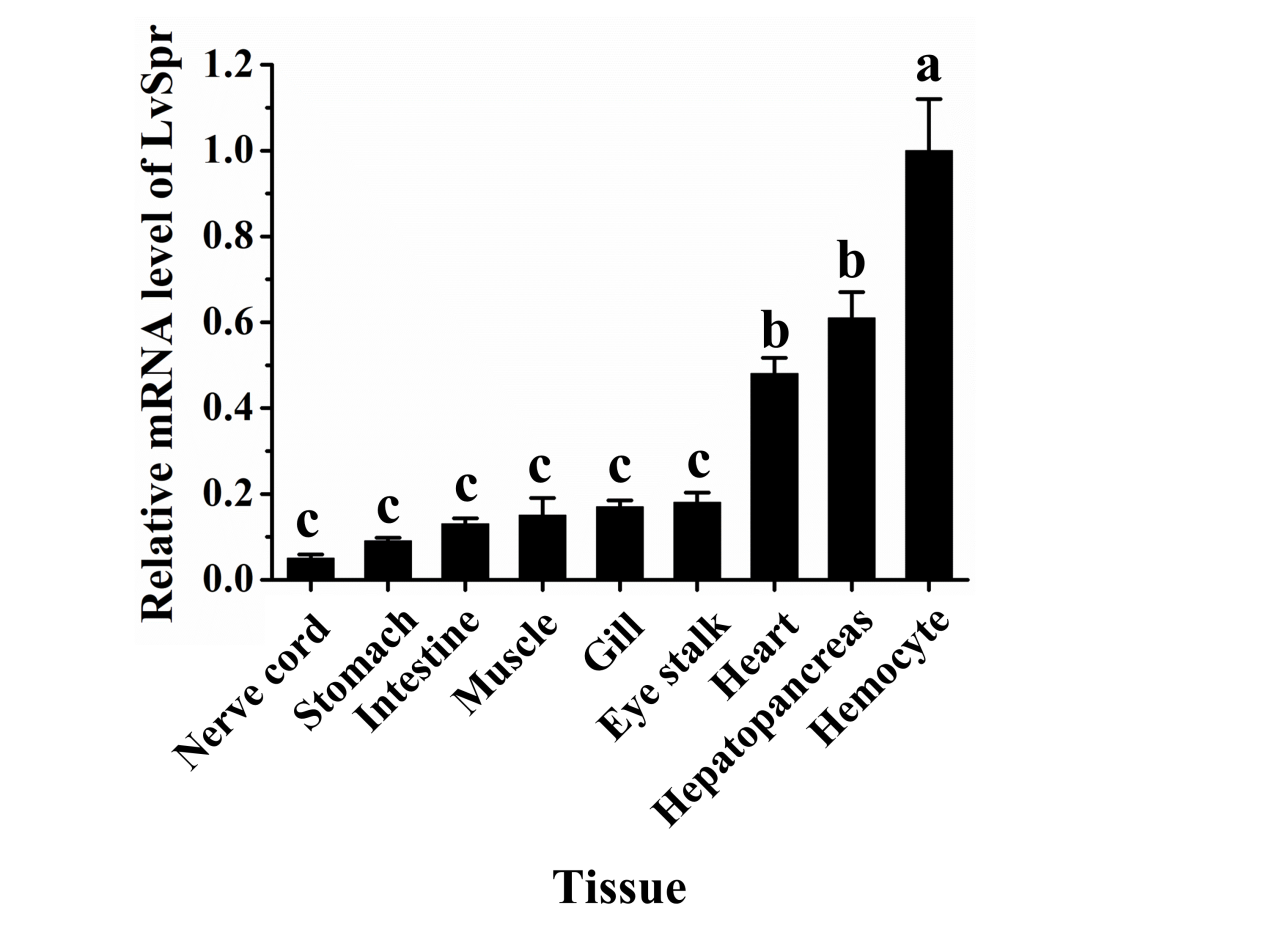


Fig. S3. Relative expression of LvSpr in different tissues of healthy*L. vannamei*. β-actin gene was chosen as internal reference for normalization. Bars represented the mean ± S.E (n=3). from three experiments. The expression of LvSpr in hemocyte was set as 1.0.Lower case letters above bars denote statistical groupings as determined by t-test following ANOVA analysis. A different lowercase letter indicates a statistically significant difference at the P < 0.05 level.Data marked with different superscripts are significantly different (*P*< 0.05).
